# Recent Advancements in the Medical Treatment of Diabetic Retinal Disease

**DOI:** 10.3390/ijms22179441

**Published:** 2021-08-31

**Authors:** Maja Szymanska, Daanyaal Mahmood, Timothy E. Yap, Maria F. Cordeiro

**Affiliations:** 1 The Imperial College Ophthalmic Research Group (ICORG), Imperial College London, London NW1 5QH, UK; maja.szymanska1@nhs.net (M.S.); daanyaal.mahmood@kcl.ac.uk (D.M.); t.yap18@imperial.ac.uk (T.E.Y.); 2 The Western Eye Hospital, Imperial College Healthcare NHS Trust (ICHNT), London NW1 5QH, UK; 3 Glaucoma and Retinal Neurodegeneration Group, Department of Visual Neuroscience, UCL Institute of Ophthalmology, London EC1V 9EL, UK

**Keywords:** diabetic retinopathy, diabetic macular edema, angiogenesis, inflammation, glycemic control, pancreatic transplantation, islet cell transplantation, anti-VEGF, corticosteroids

## Abstract

Diabetic retinal disease remains one of the most common complications of diabetes mellitus (DM) and a leading cause of preventable blindness. The mainstay of management involves glycemic control, intravitreal, and laser therapy. However, intravitreal therapy commonly requires frequent hospital visits and some patients fail to achieve a significant improvement in vision. Novel and long-acting therapies targeting a range of pathways are warranted, while evidence to support optimal combinations of treatments is currently insufficient. Improved understanding of the molecular pathways involved in pathogenesis is driving the development of therapeutic agents not only targeting visible microvascular disease and metabolic derangements, but also inflammation and accelerated retinal neurodegeneration. This review summarizes the current and emerging treatments of diabetic retinal diseases and provides an insight into the future of managing this important condition.

## 1. Introduction

Diabetic retinal disorders include a spectrum of consequences of glycemic damage in the retina, manifesting in microvascular diabetic retinopathy (DR) leading to neovascularization and retinal detachment, diabetic macular edema (DME), and accelerated underlying neurocellular degeneration [1,2]. DR is quoted as the most common microvascular complication of diabetes mellitus (DM) and a leading cause of preventable blindness in the developed world, [3] affecting approximately 95 million people worldwide [4]. The prevalence of DM is increasing and is estimated to affect 592 million people by 2035 [5]. The Diabetes Control and Complications Trial (DCCT) and the United Kingdom Prospective Diabetes Trial (UKPDS) for type 1 (T1DM) and type 2 DM (T2DM) both showed that intensive blood glucose control can delay the onset and progression of DR [6]. Yet the continual rise in patient numbers highlights the need for further improvement in treatment of DRD in order to reduce ocular morbidity. 

### Pathology of DR

DR is a microangiopathy associated with occlusion and leakage from retinal capillaries [7]. Pathogenesis involves chronic hyperglycemia, basal membrane thickening, pericyte death, and hemodynamic changes [8]. Key triggers include oxidative stress, increased expression of vascular endothelial growth factor (VEGF) and insulin-like growth factor-1 (IGF-1) [9,10]. In addition to hyperglycemia, dyslipidemia and hypertension are the major risk factors for DR [11]. However, enhanced blood pressure control does not alter DR progression compared to standard guidelines [12,13]. More recently, it has been suggested that non-alcoholic fatty liver disease (NAFLD) is a multisystem disease that could increase the risk of diabetic macro- and microvascular complications, including DR [14,15,16]. NAFLD is characterized by excessive deposition of fat in hepatocytes, not caused by excessive alcohol consumption, common in obese or diabetic patients and affects from 40.9–75% of people with T2DM [17,18]. One study demonstrated that NAFLD was linked to increased prevalence of DR and independently associated with increased prevalence PDR [19]. To the contrary, a cross-sectional study by Zhang et al. recently demonstrated that the prevalence of NAFLD in DR patients was 60.8%. Interestingly, the prevalence of DR was lower in patients with moderate (14.3%) and severe (46.6%) NAFLD, *p* = 0.024 [17]. Therefore, further research to investigate whether NAFLD increases the risk of diabetic retinopathy is needed. Better understanding of NAFLD pathophysiology could help to evaluate its relationship with DR. 

Diabetic retinopathy can be subdivided into three stages: background, pre-proliferative retinopathy, and proliferative diabetic retinopathy (PDR) characterized by neovascularization. Background DR characterizes the early stage, where chronic hyperglycemia causes damage to retinal vasculature, weakening the walls and leading to microaneurysms [7]. As diseases progresses to pre-proliferative stage, microaneurysms can eventually rupture, progressing to retinal hemorrhages. Breakdown of the blood–retinal barrier and leakage of fluid and proteins from weakened microvasculature into the retina can cause fluid deposition under the macula, thus affecting the central vision. This results in maculopathy and macular edema, the most common cause of reduced vision across all stages of DR [20]. Gradual retinal hypoperfusion leading to ischemia and loss of vascular integrity result in occlusion and degeneration of capillaries. This creates hypoxia, which stimulates the expression of proangiogenic growth factors such as VEGF. This results in abnormal growth of new vessels in the retina, ultimately leading to progression of proliferative DR. These new vessels are fragile and disorganized and, therefore, have the potential to cause vitreous hemorrhage or traction on the retina. This may eventually lead to retinal detachment. Both of these pathologies lead to potentially dramatic vision loss. 

The mechanism of how hyperglycemia leads to microvascular damage is poorly understood. Several factors and biochemical pathways have been identified to play a role in hyperglycemic damage to the microvasculature. Elevated intracellular sugar levels activate the polyol pathway, which metabolizes glucose [21]. This leads to deposition of advanced glycation end products (AGEs), activation of the protein kinase C, and upregulation of AGEs’ receptors and hexokinase pathway [22]. This results in the rise of intracellular reactive oxygen species and subsequent oxidative stress, which causes irreversible cell damage [23]. The hemodynamic changes triggered by hyperglycemia lead to vasodilation and blood flow alternations. 

Elevated glucose levels have also been shown to stimulate pericyte apoptosis, the loss of which has been implicated in the early DR stages [24,25]. Damage and loss to pericytes and endothelial cells leads to capillary occlusion and subsequent ischemia. This, in turn, stimulates hypoxia-inducible factor 1 (HIF-1), which activates the VEGF, a key mediator of angiogenesis. Other pro-angiogenic factors contributing to the pathogenesis of DR include angiopoietins (Ang-1, Ang-2), which have been recently recognized as potential novel therapeutic targets [26]. Furthermore, recent human and animal studies have suggested that the kallikrein-kinin system (KKS) may contribute to the retinal vascular permeability, vasodilation, and retinal thickening in DR [27].

Prolonged hyperglycemia and oxidative stress along with other molecular mediators drive chronic inflammation. Elevated levels of various proinflammatory cytokines such as tumor necrosis factor α (TNF-α), interleukin 1β (IL-1β), and IL-6 and chemokines such as monocyte chemoattractant protein 1 (MCP-1), CCL2, and CCL5 have been found in the DR eyes [28]. Activated cytokines secrete intracellular adhesion molecules such as ICAM-1 and VCAM-1, which attract monocytes and leukocytes and promote successive inflammatory responses [29]. Leukocytes interact with endothelial cells, inducing blood–retinal barrier breakdown and release MMPs and angiogenic factors, which further contribute to neovascularization. Improved understanding of the role of inflammation in DR pathogenesis helped to identify novel therapeutic targets, with potentially better side effects’ profile than corticosteroids.

There is also an emerging evidence that endothelial progenitor cells (EPCs) may play a role in the pathogenesis of DR [30]. EPCs are circulating, bone marrow-derived cells involved in angiogenesis and vascular homeostatis. They migrate to the site of endothelial hypoxic or ischemic injury and facilitate damage repair [31]. Hyperglycemia has been shown to result in metabolic and epigenetic alternations, subsequently affecting the angiogenic function and proliferation of EPCs. Elevated HbA1c has been associated with a decrease in the number of EPCs, whereas improved glycemic control has been linked to an increase in EPcs’ numbers [32]. In addition, EPCs obtained from patients with PDR have shown impaired vasoprotective function [33]. Furthermore, different numbers of circulating EPCs have been reported in T1DM and T2DM patients compared to healthy individuals; however, some studies have also published conflicting results [34,35,36]. Therefore, further research is needed to better understand the nuances in the relationship between EPCs and DM/DR severity. Nevertheless, EPCs are a novel therapeutic target for the treatment of DR and other microvascular diabetic complications, while also being proposed as biomarkers to highlight those patients at higher risk of proliferative disease and visual loss [37].

Retinal neurodegeneration starts early in DR progression, however, the exact underlying mechanism and its association with microvascular changes remains unclear. Inflammatory cytokines such as TNF-α and IL-1β promote apoptosis and are known to induce neuronal cell death [38,39]. Animal studies have found that apoptosis of retinal tissue in diabetic rodents was elevated and led to neuronal cell loss and retinal layer thinning. An immunohistochemistry study on diabetic human post-mortem tissue found detected the presence of pro-apoptotic molecules such as cleaved caspase-3, Bax and Fas in the nerve fiber layer and retinal ganglion cells [40]. Emerging evidence suggests, that in addition to premature neuronal death, biochemical and structural changes in neurons and glial cells also contribute to neurodegeneration [2]. Furthermore, one study found that the loss of ganglion cells and retinal cell layer preceded the microvascular changes in diabetic mice [41]. Thus, neurodegeneration could be an independent process. Further research, to better understand the molecular pathways involved in neuronal cell death and their role in the pathogenesis of DR, could help identify novel therapeutic strategies. 

The aim of this review is to summarise and comment on the established therapy, as well as the recent advancements and novel approaches in the pharmacological treatment of diabetic retinopathy and diabetic macular edema.

## 2. Ocular Treatment of Diabetic Retinal Diseases

### 2.1. Anti-VEGF Therapy

#### 2.1.1. Diabetic Macular Edema

Anti-VEGF injections are a well-established treatment for Diabetic Macular Edema, a complication of diabetes and a leading cause of blindness in diabetic patients [42]. They inhibit VEGF-driven angiogenesis and vascular permeability. There are several subtypes of VEGF, including VEGF-A/B/C/D and placental growth factor (PGF). VEGF-A is the most recognized VEGF family member involved in the pathogenesis of DRDs. Anti-VEGF-As currently and previously used for the treatment of DME include aflibercept (Regeneron, ELYEA, Tarrytown, NY, USA), ranibizumab (Lucentis; Genentech USA, Inc., San Francisco, CA, USA/Novartis Ophthalmics, Basel, Switzerland), and bevacizumab (Avastin; Genentech, San Francisco, CA, USA). They are summarized in Table 1.

**Figure 1:**
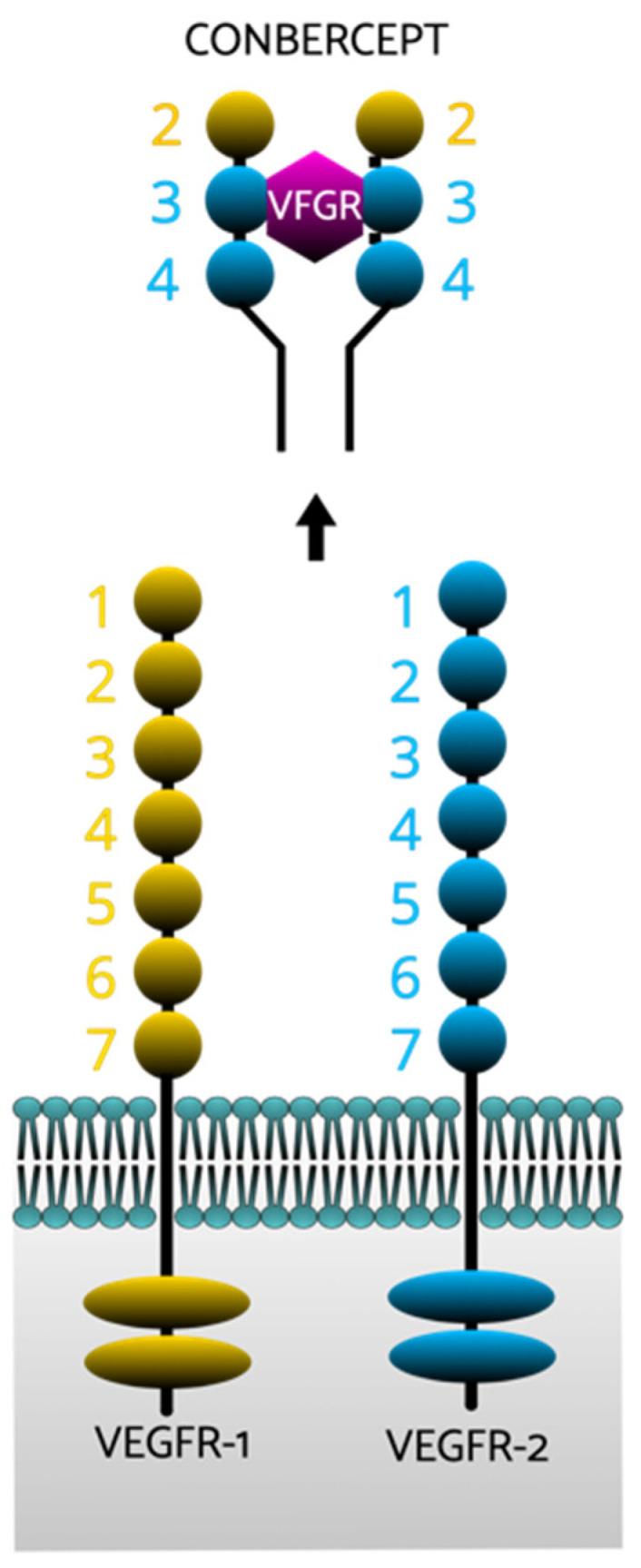
The conbercept molecule. The conbercept molecule is made up of two of the second immunoglobulins of the vascular endothelial growth factor receptor-1 (VEFGR-1) and two of the third and two of the fourth immunoglobulins of VEFGR-2 [65].

More recently, a novel anti-VEGF used for treatment of nAMD has been evaluated as a treatment for DME. Conbercept (Chengdu Kanghong Biotech, Sichuan, China) is a recombinant human VEGF receptor-Fc fusion protein with a much more potent affinity to VEGF compared to bevacizumab and ranibizumab and blocks VEGF-A/B/C isoforms and PGF (Figure 1) [64].

A meta-analysis compared the therapeutic effect of intravitreal conbercept vs. ranibizumab. Results showed no statistical difference in the mean BCVA changes, but intravitreal conbercept was superior to ranibizumab with regard to the mean CRT changes at 1, 3, and 6 months. One retrospective study compared the therapeutic efficacy of conbercept and ranibizumab for treating DME. The results of this 12-month, retrospective study showed no statistical difference in BCVA or CMT change. However, patients in the conbercept group experienced longer treatment intervals [66]. More recently, Phase I/II FRONTIER and Phase III SAILING studies showed that treatment with intravitreal conbercept or sham caused a significant improvement in the mean change in BCVA and CRT from baseline compared to sham/laser group. Furthermore, the results of the extension study showed that patients in the laser/sham group subsequently treated with conbercept monotherapy developed a significant improvement in BCVA. In summary, evidence suggests that the use of conbercept is safe and effective for the treatment of DME [67,68]. The need for a lower number of intravitreal injections than ranibizumab could improve patients’ compliance and benefit healthcare providers. Furthermore, different treatment regimens are also under investigation. The evidence from retrospective studies have shown that 3-monthly loading-dose intravitreal injections of Conbercept as well as pro re nata (PRN) treatment were efficacious in treating DME [50,69,70]. An ongoing multicenter, randomized, single-masked clinical trial will be the first to evaluate the safety and the therapeutic effect of 6 + PRN vs. 3 + PRN regimen in patients with DMO [71]. This could allow for a development of a better treatment strategy.

Brolucizumab is another novel, humanized, single-chain, monoclonal antibody fragment that inhibits VEGF-A. It is capable of a better target-tissue penetration, which could result in greater local efficacy and increased durability of the therapeutic effect [72,73]. It has already shown encouraging results in the treatment of neovascular age-related macular degeneration (nAMD) [52]. A phase III, multi-center, double-masked, randomized clinical trial that will evaluate the safety and efficacy of 4-weekly 6 mg/0.05 mL of intravitreal brolucizumab vs. 4-weekly 2 mg/0.05 mL of aflibercept in the treatment of 521 patients with DME is currently ongoing. The primary outcome of the study is the change in BCVA up to 12 months [51].

Abicipar pegol (Allergan Inc., Irvine, CA, USA) is a recombinant designed ankyrin repeat protein (DARPin) coupled to polyethylene glycol, which targets VEGF-A. It has several therapeutic advantages including high affinity, stability, and high molar concentration. It has also already shown efficacy in the treatment for nAMD in Phase III studies [74]. In addition, longer half-life and higher affinity than ranibizumab makes it an interesting therapeutic agent for DME with a potentially lower frequency of intravitreal injections [75]. Results of the open-label, multicenter, dose-escalation Phase I/II trial evaluated the safety and bioactivity of abicipar pegol. It also showed a sustained reduction in edema and an improvement in the median BCVA change in some patients. The recent interim results of the PALM study, a Phase II trial, demonstrated that over a period of 28 weeks, 2 mg of intravitreal abicipar pegol every 8 and 12 weeks showed functional and anatomical benefits with fewer intravitreal injections compared with ranibizumab administered every 4 weeks in 151 patients with DME [54].

#### 2.1.2. Diabetic Retinopathy

First-line management for PDR traditionally involves pan-retinal photocoagulation (PRP), with pars plana vitrectomy (PPV) used to treat persistent vitreous hemorrhage [76]. More recently, evidence suggests that anti-VEGF injections can be used instead of or in combination with PRP [77].

Currently, non-proliferative diabetic retinopathy (NPDR) without clinically significant macular edema requires regular monitoring with optimal blood glucose, pressure control, and lifestyle adjustments. Strategies to amend the course of DR at an earlier stage started being explored. There is increasing evidence that the use of anti-VEGF in patients with NPDR can lead to substantial regression in DR severity. The findings of the RISE/RIDE study, which investigated the treatment of DME with ranibizumab vs. sham, showed that 36% of patients in the ranibizumab group experienced a ≥2-step improvement in diabetic retinopathy severity scale (DRSS) compared to 5% in the sham group. The most prominent improvement in DR occurred in patients with moderately severe to severe NPDR at baseline [78]. These findings were similar to the results of a post hoc analysis of VIVID and VISTA studies, which investigated the intravitreal injections of aflibercept vs. laser photocoagulation for DME. It showed that 34.3% and 37.6% of patients in the aflibercept group, respectively, developed a ≥2-step DRSS improvement from baseline at week 100 [47].

PANORAMA is the first double-blinded, phase III, multicenter, prospective trial which aims to determine the effect of anti-VEGF on NPDR without DME [79]. There were 402 eyes with moderately severe to severe NPDR that were randomized into intravitreal aflibercept every 8 weeks (q8) after five loading injections, and every 16 weeks (q16) after four loading injections, and sham injection group. The completion of the study is planned for 2023; however, the recent preliminary results demonstrated significant regression of DR severity with intravitreal aflibercept compared to sham injections. At 52 weeks, 79.9% of eyes treated in q8 group and 65.2% of patients treated in q16 group improved by ≥2 DRSS steps compared to 15% of patients in the sham arm (*p* < 0.0001). Furthermore, the rate of progression to PDR was lower in aflibercept groups (3% to 3.7% vs. 20.3% in sham, *p* < 0.0001). Similarly, the proportion of eyes that developed center-involved diabetic macular edema (CI-DME) was lower in the aflibercept group (6.7% to 8.2% vs. 25.6% in sham, *p* < 0.001) [80].

Protocol W of the Diabetic Retinopathy Clinical Research (DRCR) Network, aimed to determine the role of intravitreal aflibercept vs. sham in the prevention of PDR and center involving DME in eyes with moderate to severe NPDR [81]. The recent preliminary results of a 2-year follow-up showed that aflibercept resulted in a more than 3-fold reduction in the incidence of CI-DME and a more than 2-fold reduction in the incidence of PDR in the eyes of NPDR patients. However, the preventative effect of aflibercept treatment showed no benefit on visual acuity compared to sham [82]. The results of a 4-year follow-up will be available in 2022. This will improve our understanding of optimum timing of anti-VEGF therapy in NPDR and provide more information about the long-term effect on visual acuity.

Despite the evidence, the use of anti-VEGF for the treatment of DR needs to be cautious. Patient compliance and treatment frequency as well as the effect of a therapy on the quality of life need to be considered on an individual basis. These treatments are also associated with several adverse effects, one being a 0.05% to 1% risk of endophthalmitis, which may lead to blindness [83]. Further studies are needed to determine the most efficacious duration and frequency of anti-VEGF treatment at earlier stages of DR and whether the regression in disease severity will prevent sight-threatening ocular complications. The introduction of VEGFs for the treatment of NPDR is a novel concept and more research with a longer follow-up period is needed to better determine a cost–benefit ratio of such intervention.

### 2.2. Other Anti-Angiogenic Agents

**Figure 2:**
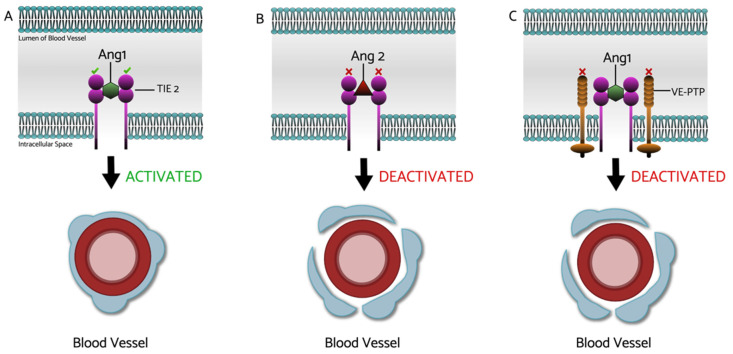
The Molecular Mechanism of Tie2/Ang. (**A**) The Binding of angiopoietin-1 (Ang1) to the tyrosine kinase 2 (Tie2) receptor is thought to enhance vascular stability by encouraging the recruitment and adhesion of peri-endothelial support cells and, as a result, allow the blood vessel(s) to maintain its normal form and function [88,89]. (**B**) The Prescence of angiopoietin-2 (Ang2), which competitively inhibits Ang1 on the Tie2 receptor, and (**C**) the presence of vascular endothelial protein tyrosine phosphatase (VE-PTP) receptors in the phospholipid bilayer are both understood to decrease the adherence of peri-endothelial support cells to the matrix and endothelial cells, thus making the blood vessel(s) more permeable and/or potentially undergo harmful angiogenesis [84,90].

Tie2 pathway regulates angiogenesis as well as vascular homeostasis and inflammation. Angiopoietins are inflammatory growth factors that bind to the Tie2 receptor, thus regulating angiogenesis. Ang1 and Ang4 are two examples of Tie2 receptor agonists (Figure 2) [84,85]. Ang2 is thought to act as a competitive inhibitor to Ang1/4; however, its role is not yet fully understood [86]. Preclinical studies have shown that Angiopoietin 1 (Ang1) plays a protective role against VEGF-induced leakage [85]. In a transgenic diabetic mouse, overexpression of Ang1 inhibited neovascularization and blood–retina barrier (BRB) breakdown. Another study on rodents demonstrated that intravitreal injections of adenovirus-expressing Ang1 inhibited leukocyte adhesion, apoptosis of retinal endothelial cells, and BRB breakdown [84]. A potential way to upregulate Tie2 activity is the inhibition of vascular endothelial phosphotyrosine phosphatase (VE-PTP). This has shown promising results in experimental studies where inhibition of VE-PTP prevented ischemia-induced retinal neovascularization [87].

Razuprotafib (AKB-9778) is a VE-PTP inhibitor that ultimately leads to Tie2 pathway activation [87]. An open-label, dose-escalation, phase I clinical trial found that subcutaneous injection of 15 mg AKB-9778 or more twice daily for 4 weeks improved BCVA in patients with DME. In addition, there was a significant correlation between improved BCVA and a reduction in CST and no adverse side effects were reported [57]. TIME-2 was a phase IIa, placebo- and sham injection-controlled, double-blinded, clinical trial that investigated AKB-9778 and ranibizumab monotherapy and combination therapy in the treatment of DME. Combination therapy achieved similar improvements in BCVA compared to the ranibizumab-only group. However, combination therapy achieved a greater reduction in CST than monotherapy. There was a small and comparable improvement in the DRSS score across all groups, with a ≥2-step change in 11.4% of all AKB-9778-treated patients compared with 4.2% in the ranibizumab monotherapy group [56]. Another study, a phase II RUBY trial, found no additional therapeutic effect of combining aflibercept with anti-Ang2 antibody, compared to aflibercept monotherapy in patients with DME [55].

Another potential therapeutic target for DME is AXT107, which promotes Tie2 pathway signaling and blocks the VEGF signaling [91,92]. AXT107 downregulates TNFα-induced inflammation in the vascular endothelial cells by converting the pro-inflammatory Ang2 into a Tie2 agonist [93]. Animal studies demonstrated that AXT107 suppressed and even caused regression of choroidal neovascularization in a laser-induced mouse model, as well as suppressed subretinal neovascularization and VEGF-induced vascular leakage in transgenic mice [92]. Therefore, AXT107 could also act as a therapeutic agent in other ocular diseases, including nAMD and retinal vein occlusion (RVO). A first-in-human clinical trial that aims to determine the safety and bioactivity of intravitreal AXT107 is ongoing with an estimated completion date in 2022 [58].

Faricimab is the first investigational bispecific antibody designed for the eye. It blocks both VEGF and Ang2 signaling pathways but does not bind to Ang1 [94]. The phase II BOULEVARD study on patients with DME investigated two doses of faricimab (1.5 mg and 6 mg) vs. monthly ranibizumab (0.3 mg) and found that the 6-mg dose of faricimab produced BCVA gains that were superior (+13.9 letters) to ranibizumab (+10.3 letters) at 24 weeks. In addition, faricimab resulted in improvements in DRSS score and dose-dependent reductions in CST, with no safety issues [60]. However, this study compared Faricimab against a low dose of ranibizumab (0.3 mg). In addition, it would be interesting to compare Faricimab with aflibercept, which has been shown to be more effective than ranibizumab in DME treatment [95]. The 1-year results of the ongoing 2-year YOSEMITE and RHINE multicenter, randomized trials showed promising results for faricimab in DME [96,97]. Both studies met their primary endpoint, with intravitreal faricimab given at up to four-month intervals, being non-inferior in visual acuity gains to intravitreal aflibercept, given every two months [59]. The estimated completion date of both studies is 2023. They will be followed by the RHONE-X long-term extension trial that will report 4-year results [98].

Nevascumab is a human IgG monoclonal antibody that binds Ang2 and blocks its binding to Tie2 [99]. Findings of the RUBY study, a phase II trial, showed no additional benefit of combination therapy with intravitreal nevascumab and aflibercept vs. aflibercept alone in patients with DME. Although the study showed some anatomic benefits in the combination therapy group, further development of Nevascumab was terminated [100].

**Figure 3:**
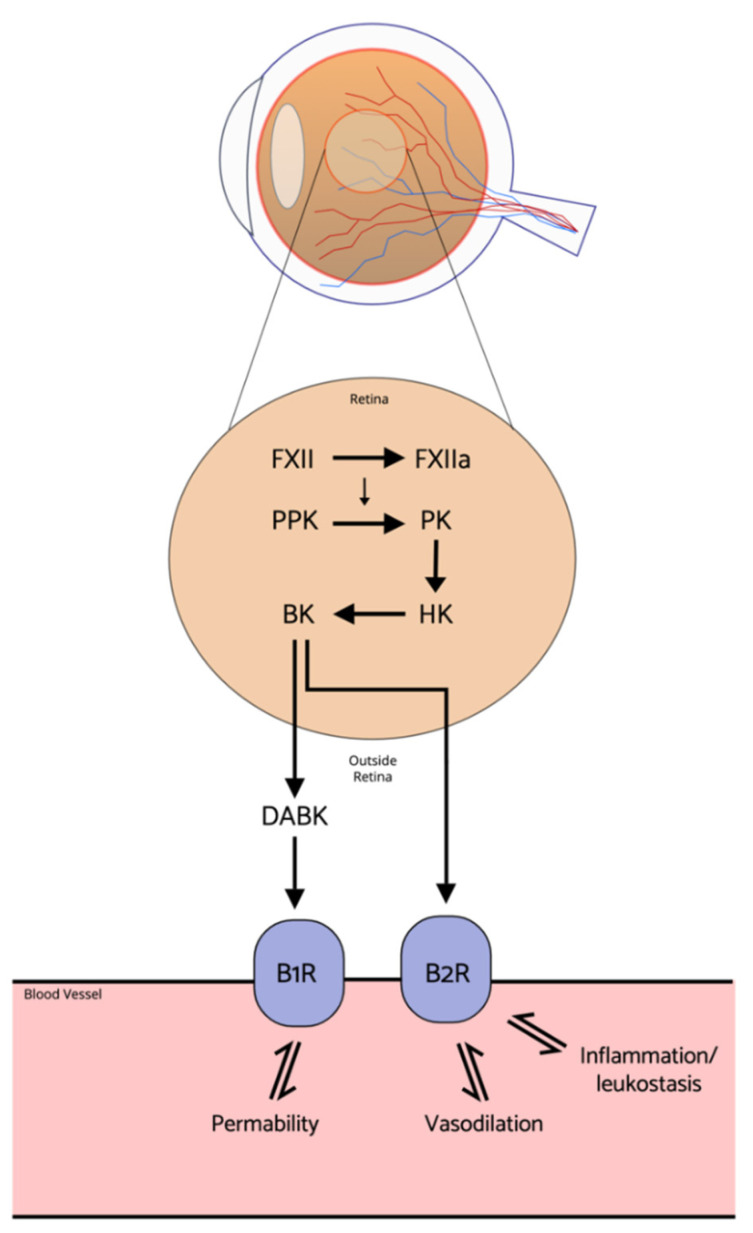
Potential action of the Kallikrein-kinin system (KKS) in DR. When the kallikrein-kinin system (KKS) is operational, Factor II (FXII) is converted and becomes Factor IIa (FXIIa) and plasma kallikrein (PK) and PK causes high-molecular-weight kininogen (HK) to separate into bradykinin (BK). BK then activates bradykinin 2 receptor (BR2) and then BK further separates into des-Arg BK (DABK), which goes on to activate bradykinin 1 receptor (BR1) [27]. Activation of these receptors leads to a downstream release of vasoactive molecules such as nitric oxide, prostacyclin, and cyclic guanosine monophosphate (cGMP), these molecules are understood to go on to cause the pathogenic effects of diabetic retinopathy [102].

The plasma kallikrein–kinin system (KKS) is another mediator of vascular permeability and retinal dysfunction recently suggested to contribute to the pathogenesis of DR and DME, independent of the VEGF pathway (Figure 3) [101]. 

KKS is known to be triggered by a vascular injury; however, mechanisms that activate the ocular KKS and their role in DR pathogenesis are not well understood. Elevated plasma kallikrein levels were found in the vitreous of patients with DME and PDR [103,104]. More recently, it has been identified as a novel therapeutic target. The KKS can be targeted in multiple ways: firstly, by enhancing the action of C1-INH, the inhibitor of PK, FXIa, FXIIa, C1r, and C1s proteases, which has already proven efficient in the treatment of hereditary angioedema [105]. An intravitreal injection of exogenous C1-INH reduced retinal vascular hyperpermeability in a diabetes-induced rodent model [106].

Selective PK inhibition is another potential strategy that could downregulate the pro-inflammatory action of KKS. Rodent studies have shown that intravitreal injection of ASP-440, a PK inhibitor, improved ocular blood flow and reduced vascular permeability [106]. Other foci of interest are B1 and B2 receptors, which are widely expressed in the eye and are activated by both KKS and PK pathways. Several preclinical studies on rodents showed that locally and systemically administered B1R antagonists effectively reduced vascular permeability [107,108].

KVD001 (KalVista Pharmaceuticals, Cambridge, Massachusetts, U.S) is a selective plasma kallikrein inhibitor, which has the potential as an IVT therapy for DME. A sham-controlled, double-masked, phase II clinical trial evaluated two doses (6 μg and 3 μg) of intravitreal KVD001 for treatment of persistent macular edema [109]. The results of the study showed no statistically significant difference in BCVA, CST, or DRSS at either dose compared to sham and no safety issues. However, there was some evidence of a protective effect against vision loss. Of patients treated with 6 μg of KVD001, 32.5% lost their vision compared to 54.5% of patients in the sham group (*p* = 0.042) [61]. Further studies are needed to determine the role of kallikrein inhibitor in preserving vision in DME patients and its therapeutic effect. KalVista currently aims to develop an oral plasma kallikrein therapy for DME [61]. This could revolutionize the treatment of DME and reduce the burden associated with intravitreal injections. Several other drugs targeting plasma kallikrein are under investigation. One of them is THR-149 (Oxurion, Iselin, New Jersey, U.S), a kallikrein bicyclic peptide inhibitor that has recently been trialed in a phase I, open-label, multicenter, non-randomized study in 12 DME patients [110]. Findings show that following a single intravitreal THR-149 injection, there was rapid improvement in the mean BCVA (3.9 letters on day 1, 7.5 letters on day 14), which was preserved until the end of the study on the 90th day [62]. Further clinical investigations are planned. 

Promising preclinical studies along with an encouraging safety profile from phase I studies provided hope for the development of a novel monotherapy or combination therapy for DME that will target a VEGF-independent pathway. However, the targeting of Ang/Tie2 in clinical studies has been unsuccessful in improving BCVA or reducing CRT. Different doses, treatment frequency, and delivery methods should be explored. Further research and a better understanding of Ang/Tie2 could help identify other targets.

The Rho/Rho kinase (ROCK) pathway has been shown to promote leukocyte adhesion by affecting the expression of Intercellular Adhesion Molecule-1 (ICAM-1), integrins, and other molecules, leading to endothelial damage [111]. Evidence suggests that the ROCK pathway is involved in the pathogenesis of DR and DME as well as VEGF-induced angiogenesis [112]. In ophthalmology, ROCK inhibitors have been used for the treatment of glaucoma [113]. More recently, the potential for the treatment of DR and DME has been explored. A randomized clinical trial by Ahmadieh et al. compared monthly intravitreal injections of bevacizumab alone vs. a combination with intravitreal fasudil for 3 months in 44 eyes with center-involving DME. Results showed that the combination therapy was superior to bevacizumab alone, with a statistically significant improvement in BCVA at 3 and 6 months [63]. However, the follow-up of this study was short. ROCK inhibitors have also been associated with conjunctival hyperemia [114]. Therefore, more studies are needed to further validate their clinical use in DR and DME. In addition, delivery of ROCK inhibitor requires frequent intravitreal injections, due to its short half-life. A solution to that could be intravitreal implantation [111].

### 2.3. Corticosteroid Therapy

Corticosteroids target pro-inflammatory mediators involved in DME, including IL-6, IL-8, MCP-1, ICAM-1, TNF-α, HGF, and ANGPT2 as well as reduce VEGF synthesis. They also decrease the production of thromboxanes, leukotrienes, and prostaglandins, thus improving the activity of tight junctions in retinal vessel endothelium. Intravitreal corticosteroids are an alternative treatment option for DME to laser and anti-VEGFs. However, due to their side effects, including a rise in intraocular pressure (IOP) and cataract, they have a secondary role to anti-VEGF injections. They can be used in cases of refractory DME or if there is an insufficient response to anti-VEGFs. The most commonly used corticosteroids include triamcinolone acetonide, dexamethasone, and fluocinolone acetonide They are summarized in Table 2.

A randomized, multicenter, masked, phase III clinical trial investigated the dexamethasone implant vs. sham for treatment of DME. Throughout the 3 years, patients in the DEX implant groups (0.7 and 0.35 g) had a significantly greater improvement in BCVA compared to sham group (22.2, 18.4, and 12.0%, respectively, *p ≤* 0.018). However, cataract developed in over 60% of patients in the DEX implant group [118]. In addition, results of a pooled analysis showed that both 0.35- and 0.7-mg implant doses delayed 2-step DR worsening by 12 months [125]. Another prospective, multicenter, clinical trial investigated a fluocinolone (FA) implant (Retisert) in patients with persistent or recurring DME vs. standard of care (laser or observation). Results showed that among patients who received 0.59-mg FA implant, VA improved ≥3 lines in 16.8% of eyes at 6 months (*p* = 0.0012; SOC, 1.4%). However, a 3-year follow-up showed no statistically significant difference between the FA implant group (31.1%) and standard care group (20.0%; *p* = 0.1566). In addition, the proportion of patients in the FA implant group who developed a rise in IOP and cataract was high, 61.4% and 91%, respectively [126].

The fluocinolone acetonide implant (Iluvien) containing lower doses (0.23 and 0.45 μg) has also been tested. A study compared 0.23 and 0.45 μg Iluvien vs. sham in patients with persistent DME. At the 3-year follow-up, it was found that the proportion of patients who gained at least 15 in letter score, using the last observation carried forward method, was 28.7% and 27.8%, respectively, compared to 18.9% in sham patients (*p* = 0.018). In addition, an improvement in at least 2 steps in the DRSS scale occurred in 13.7% (0.23) and 10.1% (0.45) compared with 8.9% in the sham group [127]. More recently, 2-year interim safety results of the observational PALADIN study showed a favorable benefit–risk profile for the 0.2 µg/day fluocinolone acetonide intravitreal implant for the treatment of diabetic macular edema in previously steroid-challenged patients [128]. 

Given the efficacy of anti-VEGF and corticosteroids in the treatment of DME, a potential for combination therapy was explored. Protocol U from DRCR Network was a phase II clinical trial that showed no significant benefit of adding dexamethasone implant to ranibizumab injections for persistent center-involving DME. Similarly, recently published results of a randomized, double-blinded study showed similar 24 weeks’ BCVA outcomes between patients who received a combination of triamcinolone acetonide and intravitreal aflibercept vs. aflibercept monotherapy [129].

Alternative corticosteroid delivery strategies have been under investigation. One of the recent technological advancements includes suprachoroidal drug delivery, which can achieve up to 10 times greater chorioretinal concentration compared to intavitreous route. This way of drug administration also reduces the exposure to vitreous and anterior segment, thus potentially having a better side effects’ profile. The recent results of the HULK study, a phases I and II exploratory clinical trial, demonstrated that suprachoroidal triamcinolone acetonide (SCTA), in a form of monotherapy and combination with intravitreal aflibercept, showed an increased efficacy and durability, with no safety concerns [130]. Evidence suggests that SCTA is well tolerated and may improve functional and anatomical outcomes among treatment-resistant DME patients [131].

### 2.4. Other Anti-Inflammatory Agents

Tumor Necrosis Factor alpha (TNF alpha) is an acute inflammatory cytokine produced by macrophages involved in signaling during necrosis or apoptosis [132]. Increased serum levels of TNFα have been found in the vitreous fluid of diabetic patients [133]. In addition, there is a strong correlation between plasma levels of TNF-α and severity of DR [134]. Anti-TNF-α therapy has shown some promising results in animal studies. Subcutaneous injection of Etanercept, a TNFα inhibitor, significantly reduced ocular inflammation and vascular leakage in diabetic rodents [135]. A pilot study on seven patients investigated the use of Etanercept in the treatment of refractory DME and found no significant improvement in BCVA. Although this study included only seven patients, there was no strong evidence of therapeutic efficacy of etanercept in DME treatment [120]. 

Infliximab and Adalimumab, TNFα-neutralizing antibodies, have also been investigated for the treatment of DME. However, a study by the Pan-American Collaborative Retina Study Group showed no functional or anatomical benefit of intravitreal infliximab or adalimumab in patients with refractory DME [121]. Furthermore, a randomized, double-blinded, placebo-controlled, crossover study showed some improvement in ETDRS scores in 13 patients with persistent DME; however, at 32 weeks the improvement in BCVA from baseline in the placebo group was greater than in the infliximab group. There was also no effect of infliximab on anatomic changes [136]. Thus far, there is no evidence showing the benefit of infliximab on the treatment of DME. In addition, there are concerns about the adverse effects of the therapy, such as infections or autoimmune disorders, due to the need for administering high concentrations of Infliximab (5 mg/kg) multiple times [137]. 

Experimental DR models have identified CCR2 and CCR5 chemokines, which are expressed on the surface of monocytes. They are involved in signaling pathways associated with retinal vascular leakage, macrophage infiltration, and angiogenesis [138]. CCR2 has also been associated with regulating VEGF production. Their levels were also found to be elevated in the vitreous and aqueous humor of patients with diabetic retinopathy and DME [139]. Therefore, antagonism of CCR2 and CCR5 could help reduce the inflammation and vascular leakage in the diabetic retina. A randomized, placebo-controlled, double-masked, phase II trial by Gale et al. investigated PF-04634817, a CCR2/CCR5 dual antagonist for treating DME. This study found that its therapeutic effect was inferior to monthly intravitreal injections of ranibizumab [123]. 

Research is also focused on targeting integrins involved in leukostasis, the early step of inflammation. The DEL MAR phase II trial investigated the therapeutic effect of integrin inhibitor ALG-1001 (Luminate, Allegro Ophthalmics) as sequential therapy to a single anti-VEGF injection in DME patients compared to anti-VEGF monotherapy. The study found that the mean gain in BCVA was 7.1 letters for patients in the ALG-1001 with bevacizumab pre-treatment group compared to 6.7 letters for patients in the bevacizumab control group [124]. Therefore, ALG-1001 has the potential as an alternative form of DME treatment with fewer intravitreal injections. Furthermore, it could provide hope for patients unresponsive to anti-VEGF therapy.

### 2.5. Targeting Neurodegeneration

The principles of preventing retinal neurodegeneration involve targeting dysregulated metabolic pathways and control of blood pressure [140]. More recently, the use of neuroprotective agents in the treatment of DR has been explored. Elevated levels of glutamate have been found in the retina and vitreous of diabetic patients. Evidence suggests that excess glutamate may lead to excitotoxicity and damaged retinal neurons [141]. High concentrations of extracellular glutamate activate N-methyl D-Aspartate (NMDA) receptors, leading to neuronal cell depolarization, rise in intracellular calcium levels, and subsequent free radical production and apoptosis [142]. Preclinical studies have shown that intravitreal injection of MK-801, (N-methyl D-aspartate (NMDA) receptor antagonist), Memantine (Glutamate receptor antagonist), and Pentazocine (specific sigma receptor-1 ligand) showed neuroprotective properties in the retina of diabetic rodents [143,144]. Furthermore, other experimental studies showed that known neuroprotective factors such as BDNF, NGF, PEDF, VEGF, Insulin, and Epo protected neurons in animal models of diabetic retinopathy [145,146,147,148]. 

Cibinetide (ARA 290, Araim Pharmaceuticals), erythropoietin (EPO)-derived peptide, has been shown to reduce retinal thinning and inflammation [149]. Due to its promising neuroprotective properties, ARA290 has been recently investigated as a treatment for DME. A Phase II Clinical Trial that will evaluate the use of Cibinetide for the Treatment of DME is currently ongoing. The main outcome measure is the mean change in BCVA from baseline up to 12 weeks after daily 4 mg of subcutaneous cibinetide in patients with DME [150]. 

Lowering oxidative stress could be another therapeutic strategy in diabetic retinal neurodegeneration. Several animal studies suggest that antioxidants have anti-inflammatory and neuroprotective properties in experimental diabetic retinopathy [151,152,153]. However, a clinical study with a 5-year follow-up demonstrated that the effect of oral antioxidant administration in T2DM patients with NPDR had no effect on the BCVA [154].

Lutein, a naturally occurring carotenoid and a potent antioxidant, has been shown to inhibit oxidative stress and maintained the thickness of the retinal neural layer, thus suggesting that lutein may have a neuroprotective role in diabetic eyes [153]. Lutein treatment has also been associated with reduced gliosis and reduced production of pro-inflammatory factors from Müller cells [155]. More recently, a placebo-controlled clinical trial reported by Zhang et al. investigated the effect of lutein on diabetic patients with DR. A 36-week follow-up showed no statistically significant visual improvement. However, contrast sensitivity increased at specific low frequencies [156].

Brimonidine is a selective alpha2-adrenergic receptor agonist used for the treatment of glaucoma. It reduces intraocular pressure and increases ocular perfusion, protecting the retinal ganglion cells from pressure-induced ischemia and subsequent apoptosis. Furthermore, it has been shown to block glutamate excitotoxicity-induced oxidative Stress in ischemic retinal injury [157]. Brimonidine administered at the early stage of NPDR has been shown to reduce retinal ischemia in patients with well-controlled, long-standing T2DM. Improved visual acuity and decreased micro-aneurysm formation were also reported [158]. A prospective, randomized, controlled trial including 30 eyes evaluated topical brimonidine as a treatment option for clinically significant macular edema with ischemic changes in diabetic maculopathy. Results showed that brimonidine had no significant effect on retinal ischemia or visual acuity. More recently, results of the EUROCONDOR, a 96-week, randomized, prospective, phase II/III, multicenter trial were published [159]. This study assessed whether the topical administration of brimonidine and somatostatin could prevent retinal neurodysfunction in T2DM patients. The results did not demonstrate any neuroprotective properties of either agent; however, venular dilation in patients with pre-existing early DR was observed in both treatment groups, compared to placebo [160]. Therefore, the authors suggest that neuroprotective agents could be useful in preventing the worsening of pre-existing retinal neuro-dysfunction.

## 3. Other Emerging Ocular Treatments

### 3.1. THR-149 and THR-687

THR-687 is a novel pan RGD (arginylglycylaspartic acid) integrin antagonist. Activation of RGD receptors promotes vascular leakage, angiogenesis, inflammation, and fibrosis [161]. Therefore, THR-687 has the potential to amend the course of DR, independent of anti-VEGF responsiveness. Preclinical studies showed that THR-687 inhibited vascular leakage in a mice model and neovascularization-induced leakage in a monkey [161]. A recent phase I open-label study evaluated the safety profile of THR-687, and Phase II will investigate it as a VEGF-independent treatment option in DME patients [162].THR-149 is a reversible plasma kallikrein inhibitor, the role of which in the pathogenies of DR was described in the previous section [161]. The Phase I clinical trial showed that all investigated doses (0.005 mg, 0.022 mg, and 0.13 mg) of THR-149 were safe and tolerated [163]. A Phase II randomized clinical trial (KALAHARI) will investigate THR-149 as a treatment for DME. The planned completion date is in 2023 [110]. 

### 3.2. OPT-302

OPT-302 (sVEGFR-3) is a novel biologic drug that neutralizes VEGF-C and VEGF-D pathways [164]. Therefore, when combined with the anti-VEGF-A drugs, it has potential as a new therapy for those who are resistant to current forms of anti-VEGF-A treatment. Recent results of a randomized, double-masked, dose expansion Phase IIa clinical trial showed that a combination therapy of intravitreal OPT-302 with aflibercept in patients with refractory DME resulted in a gain ≥ 5 letters of visual acuity at week 12 in 52.8% patients (*n* = 115) with refractory DME, with no safety concerns [165]. However, further studies with a longer follow-up will need to determine the long-term efficacy of this combination therapy as well as examining the functional and anatomical endpoints.

### 3.3. GB-102

GB-102 (sustained-release sunitinib malate) inhibits multiple intracellular tyrosine kinases (RTKs), being a potent inhibitor of VEGFR-1, -2, and -3. It is administered via an intravitreal injection in formulation that aggregates to a depot and subsequently breaks down into lactic acid and glycolic acid [166]. This could allow for a significantly reduced frequency of intravitreal injections and hospital visits. The multicenter, open-label, parallel-arm, Phase IIa trial evaluated the safety, tolerability, and pharmacodynamics of a single intravitreal injection of GB-102 at two doses (1 mg and 2 mg) with a 6-month follow-up [167]. However, the results are not available yet. The ALTISSIMO trial reported no safety and tolerability issues in patients with neovascular age-related degermation treated with GB-102 [168]. Apart from the long-term efficacy of such therapy, the rate of particle dispersion and migration in the anterior chamber as well as treatment frequency and a potential for a combination therapy should be investigated [169].

## 4. Systemic Treatment of Diabetic Retinopathy and Islet Cell Transplantation

Systemic treatment is another key component of DR prevention and management. It involves adequate glycemic, blood pressure, and lipid control, as well as lifestyle adjustments [170].

A major advance in diabetic management is the introduction of pancreas transplants. Vascularized pancreas transplants are an established treatment in T1DM and are increasingly used in T2DM with low insulin production [8,171]. The majority of patients experience immediate normalization or reduction in blood glucose concentration after successful transplantation and are less likely to experience episodes of severe hypoglycemia [172]. Other beneficial effects of successful pancreatic transplant include the regression of atherosclerosis, normalization of diastolic and systolic heart function, and reduction in cardiovascular events [173,174,175]. However, the impact on microvascular complications (retinopathy, nephropathy, and neuropathy) remains less clear [176].

Several studies have attempted to evaluate the changes in DR following pancreatic transplant, reporting a variety of findings including deterioration, improvement, and stabilization [177,178,179,180]. These studies are difficult to compare due to variation in follow-up, population characteristics, and retinopathy grading systems. The findings of the most recent and largest study (*n* = 303) by Yoon Jeon et al. showed that 20.5% of eyes showed a DR progression in a mean follow-up of 4.2 years. However, similarly to other studies, a significant proportion of the cohort (221 eyes, 72.9%) had already been diagnosed with advanced DR and had a history of PRP or PPV prior to transplant [181]. This can be explained by the fact that pancreatic transplants are offered to long-term diabetic patients who have already developed DM complications; thus, it has only been possible to study the effects of transplant in an advanced cohort of eyes [172]. 

Early DR worsening has previously been observed in 10–20% of patients within 6 months following pancreas transplant or bariatric surgery [182]. A study looking at DR after combined kidney–pancreas transplantation showed early worsening (defined as 2.3 years following transplantation), characterized by vitreous hemorrhages and increased risk of cataract [183]. More recently, a sub-analysis of 43 patients from the ongoing randomized clinical study EMA-SPK, which will evaluate the differences in retinal neovascularization in patients with DR, has shown that 16 patients (37.2%) developed an early worsening of diabetic retinopathy within 12 months following a pancreas and kidney (PAK) transplant [184]. Several theories exist explaining why retinopathy may not remain stable following a pancreas transplant. Ischemia and hypoxic conditions in the diabetic retina are implicated in the “VEGF hypothesis”; in vitro experiments on human cells have shown that under hypoxic conditions, cells increase reliance on glucose as their main energy source. When the energy supply becomes restricted due to sudden normoglycemia, cells then upregulate VEGF, which may lead to the progression to proliferative disease [185]. Another explanation of these findings could be a phenomenon called “metabolic memory” [186], with studies showing that adverse effects of hyperglycemia, including oxidative stress, advanced glycation end products, and epigenetic changes, can persist despite achieving satisfactory glycemic control [187,188,189,190]. 

Newer methods of transplantation such as islet cell transplants are becoming available and have been carried out in the United Kingdom National Health Service since 2008 [191]. Compared to pancreas transplantation, islet cell transplantation is less invasive, has lower morbidity, and allows for storage of the islet graft in tissue culture or cryopreservation for banking [192]. One prospective, crossover study compared the effect of intensive medical therapy vs. islet cell transplantation on the progression of diabetic retinopathy. A mean 36-month follow-up period revealed that six out of 51 eyes experienced a DR progression compared to zero in the islet transplant group (*p* < 0.02). However, this study was not randomized or masked. Furthermore, a meta-analysis that included randomized, controlled trials and controlled cohort studies reported that DR progression was reduced in patients who received an islet cell transplantation vs. medical therapy (RR 0.25; 95% CI 0.08–0.71) [193]. However, islet cell transplantation has several limitations, such as the use of immunosuppressive agents, complications of which include mouth ulcers, diarrhea, and acne in the short term and malignancy and infections in the long term [194]. In addition, a major problem is the occurrence of instant blood-mediated inflammatory reaction (IBMIR), the main cause of tissue loss [195,196]. Rarely, it can lead to portal vein thrombosis, hepatic infarction, and portal hypertension [197]. Novel ways of islet cell transplantation are being tested to improve islet graft survival. The anterior chamber of the eye (ACE) is a potential new site for therapeutic islet transplantation because of its high oxygen tension and immune-privileged properties. A recent study involving a nonhuman primate with induced T2DM showed that transplantation of islets into the ACE improved the glycemic parameters and reverted the progression of diabetes [198]. The results of pre-clinical studies are promising. Further research is needed to investigate the treatment effect on the retina and determine whether this will be replicable in humans [199,200]. 

A large prospective study with a control group and a longer follow-up period is needed to establish the effect of pancreatic or islet cell transplantation on diabetic retinopathy. However, for the majority it does appear that successful pancreatic transplants are universally associated with the stability of DR. Future work should focus on the development and use of imaging-based biomarkers that are able to further stratify DR in order to reliably identify peri-operative factors that may improve functionally significant visual outcomes.

## 5. Cell-Based Therapy

Over the past years, stem cell transplantation has been of major interest in the treatment of DRDs. The use of stem cells could allow for regeneration of atrophic and damaged retina and potentially reverse the disease process [201]. Preclinical studies have investigated several different cell types as a potential novel therapy. These involve Embryonic or induced pluripotent stem cells (iPSC), mesenchymal stem cells, and vascular precursor cells such as hematopoietic cells or endothelial progenitor cells [202].

Mesenchymal stem cells have several advantages as potential DR therapy such as being easily harvested from a variety of sites, including bone marrow, placenta, or adipose tissue, as well as being capable of secreting neurovascular protective paracrine factors [203]. Preclinical studies demonstrated that intravenous administration of MSCs in diabetic rodent model resulted in improved glucose levels and blood–retinal barrier [204]. Other sites of administration have been explored such as intravitreal and subretinal injections [205]. Emerging evidence that EPCs may be implicated in the pathogenesis of DRDs revealed a novel therapeutic target [30]. Animal studies have shown that EPCs positive for CD34 marker are mobilized in response to hypoxia, enter the circulation, and are capable of differentiating into endothelial cells and promoting vascular repair. Both intravenous and intravitreal administration of CD34+ cells in murine models have shown promising results [204].

Clinical trials are currently investigating stem cell therapy for DR in the USA. A pilot clinical trial, which will determine the safety of intravitreal autologous adult bone marrow CD34+ stem cells in patients with irreversible blindness caused by a variety of retinal disorders, including DR, is currently ongoing. This study will involve 15 participants with a follow-up period of 6 months [206]. Another observational cohort study will aim to harvest human inducible pluripotent stem cells (iPSCs) from peripheral blood and differentiate iPSCs into CD34+ cells and mesoderm, which will then be intravitreally injected into diabetic rodents and primate eyes. This will help to determine whether iPSCs are capable of differentiating into endothelial and pericyte cells in diabetic eyes, and thus act as a potential therapeutic strategy [207]. 

Although the use of stem cell therapy could revolutionize the management of DR, its therapeutic application is still in early stages. Further research needs to be conducted prior to implementing stem cell therapy into clinical practice. Some of the challenges include determining the safety, efficacy, and durability of stem cell therapy, and number and type of cells needed as well as the therapeutic window and target patient population [201].

## 6. Conclusions

This review summarized both the well-established pharmacological treatment options as well as recent developments, novel therapeutic agents, and emerging therapies that have the potential to revolutionize the management of DR and DME. With the ability to improve visual function and prevent visual loss, anti-VEGF therapy has significantly improved the care and prognosis of DR patients. Its role in the treatment of NPDR could aggressively treat DR at an earlier stage and prevent sight-threatening complications. However, anti-VEGFs have several limitations such as a need for frequent injections, possible treatment resistance, potential side effects, and costs. Clinical trials evaluating the novel anti-angiogenic agents targeting VEGF-independent pathways show promising results and potential for combination therapy. However, further research is needed to determine their long-term efficacy and cost–benefit ratio. Corticosteroids are an alternative option for patients with refractory DME or insufficient response to anti-VEGF. The development of intravitreal implants has allowed for less frequent intravitreal injections; however, their side effect profile favors the use of anti-VEGFs as first-line therapy. The discovery of the role of inflammation and neurodegeneration in DR pathogenesis has revealed several molecules that can act as potential therapeutic targets. However, the translation of many novel therapeutic strategies from experimental studies to clinical trials has failed. Thus, further studies are still needed to better understand the exact molecular mechanisms underlying their role in DR. Further research of the novel drugs needs to determine the treatment frequency, duration, and the potential for combination therapies as well as how to effectively translate them into clinical practice.

## Figures and Tables

**Table 1:** Anti-angiogenic agents for treatment of DR and DME.

Drug	Mechanism of Action	Administration Route	Ophthalmic Outcomes	Possible Side Effects
Ranibizumab [43,44]	Anti-VEGF	Intravitreal	Superior BCVA improvement and greater reduction in CRT compared to laser in treating DME Non-inferior to PRP in treating PDR DR improvement in patients with NPDR	Rise in the IOP Vitreous hemorrhage Inflammation
Pegaptanib [45]	Anti-VEGF	Intravitreal	Significant improvement in BCVA vs. sham in treating DME	Rise in IOP Conjunctival hemorrhage
Aflibercept [46,47]	Anti-VEGF	Intravitreal	Superior in improving BCVA vs. laser in treating DME and PDR	Rise in IOP Vitreous hemorrhage Inflammation
Bevacizumab [48]	Anti-VEGF	Intravitreal	Superior in reducing in CRT and improving visual acuity compared to laser	Rise in IOP Vitreous hemorrhage Inflammation
Conbercept [49,50]	Anti-VEGF	Intravitreal	Significantly improves BCVA compared to baseline in treating DME Non-inferior to ranibizumab in treating DME	Rise in IOP Conjunctival hemorrhage
Brolucizumab [51,52]	Anti-VEGF	Intravitreal	Phase III trial ongoing	Conjunctival hemorrhage eye pain conjunctival hyperemia
Abicipar pegol [53,54]	Anti-VEGF	Intravitreal	Functional and anatomical benefits with fewer intravitreal injections compared with ranibizumab in patients with DME	Inflammation Vitreous/Conjunctival hemorrhage Vitreous floaters
Razuprotafib (AKB-9778) [55,56,57]	VE-PTP inhibitor	Intravitreal	Significant improvement in BCVA from baseline in treating DME Combination therapy with ranibizumab achieved a greater reduction in CST than a monotherapy, but similar improvement in BCVA No significant improvement in DRSS score in NPDR patients compared to placebo	Dizziness Headache
AXT107 [58]	Tie2 activator Anti-VEGF-A Anti-VEGF-C	Intravitreal	Phase I trial in progress	To be clarified
Faricimab [59,60]	Bispecific antibody; VEGF and Ang2 inhibitor	Intravitreal	Superior to ranibizumab in improving BCVA in DME patients at 24 weeks Improves DRSS score and dose-dependent reductions in CST Non-inferior in visual acuity gains to intravitreal aflibercept in treating DME	Rise in the IOP Vitreous hemorrhage Inflammation
KVD001 [61]	plasma kallikrein inhibitor	Intravitreal	No statistically significant improvement in BCVA, CST, or DRSS compared to sham in treating DME. Some evidence of protection against vision loss.	Rise in IOP Inflammation
THR-149 [62]	plasma kallikrein inhibitor	Intravitreal	Rapid improvement in the mean BCVA from baseline in DME patients.	To be clarified
Fasudil [63]	ROCK inhibitor	Intravitreal	Significant improvement in BCVA in a combination therapy with intravitreal bevacizumab vs. bevacizumab alone.	Conjunctival hyperemia, Subconjunctival hemorrhage Cornea verticillata

**Table 2:** Anti-inflammatory agents for the treatment of DME.

Drug	Mechanism of Action	Administration Route	Ophthalmic Outcomes	Possible Side Effects
Triamcinolone [115]	Corticosteroid	Intravitreal Topical Periocular	Greater improvements in BCVA when combining triamcinolone with prompt laser vs. laser alone in pseudo-phakic eyes with DME.	Cataract Rise in IOP Vitreous hemorrhage
DEX Implant (Ozurdex) [116,117,118]	Corticosteroid	Intravitreal implant	Greater improvement in BCVA and greater reduction in CRT compared to sham in patients with DME.	Cataract Rise in IOP Vitreous hemorrhage
Fluocinolone Acetonide (Iluvien) [119]	Corticosteroid	Intravitreal insert	Greater improvement in BCVA compared to sham group in treating DME	Cataract Rise in IOP Glaucoma
Etanercept [120] Infliximab [121] Adalimumab [121,122]	TNFα inhibitor	Intravenous Intravitreal	No significant functional or anatomical benefits in DME treatment.	Uveitis Inflammation Infections Autoimmune disorders Demyelination Malignancies Heart failure
PF-04634817 [123]	CCR2 and CCR5 inhibitor	oral	Modest improvement in BCVA, but inferior intravitreal ranibizumab therapy for DME.	To be clarified
ALG-1001 (Luminate) [124]	Anti-integrin	intravitreal	Improved BCVA when used in combination with bevacizumab vs. bevacizumab alone in patients with DME. Needs fewer intravitreal injections compared to bevacizumab.	To be clarified

## Data Availability

Not applicable.
